# Multi-omic assessment of mRNA translation dynamics in liver cancer cell lines

**DOI:** 10.1038/s41597-025-05861-5

**Published:** 2025-08-30

**Authors:** Asier González, Muskan Pandey, Niels Schlusser, Sayanur Rahaman, Meric Ataman, Nitish Mittal, Alexander Schmidt, Attila Becskei, Mihaela Zavolan

**Affiliations:** 1 Biozentrum, University of Basel, Spitalstrasse 41, 4056 Basel, Switzerland; 2 Departament de Bioquímica i Biologia Molecular and Institut de Biotecnologia i Biomedicina, Universitat Autònoma de Barcelona, 08193 Cerdanyola del Vallès, Spain; 3 Institute of Molecular Biology and Biophysics, Department of Biology, ETH Zurich, 8093 Zurich, Switzerland; 4 Swiss Institute of Bioinformatics, Basel, Switzerland

**Keywords:** Time series, Bioinformatics

## Abstract

The limited correlation between mRNA and protein levels within cells highlighted the need to study mechanisms of translational control. To decipher the factors that determine the rates of individual steps in mRNA translation, machine learning approaches are currently applied to large libraries of synthetic constructs, whose properties are generally different from those of endogenous mRNAs. To fill this gap and thus enable the discovery of elements driving the translation of individual endogenous mRNAs, we here report steady-state and dynamic multi-omics data from human liver cancer cell lines, specifically (i) ribosome profiling data from unperturbed cells as well as following the block of translation initiation (ribosome run-off, to trace translation elongation), (ii) protein synthesis rates estimated by pulsed stable isotope labeled amino acids in cell culture (pSILAC), and (iii) mean ribosome load on individual mRNAs determined by mRNA sequencing of polysome fractions (polysome profiling). These data will enable improved predictions of mRNA sequence-dependent protein output, which is crucial for engineering protein expression and for the design of mRNA vaccines.

## Background & Summary

Translation is the mRNA-mediated process of decoding the genetic information to generate proteins^1^. Regulatory mechanisms operating at this level^2,3^ result in widely varying rates of protein synthesis across mRNAs^4^, subcellular locations and time^5^, while dysregulation of translation leads to disease^6^ including cancer^7^. Translational control is prominent in stem cells^8^ and during development^9,10^, upon cell proliferation and differentiation^11^, as specialized cell types such as T cells carry out their functions^12^, and under cellular stress^13^. The sequence of 5′ and 3′ untranslated regions (UTRs), their interactions with RNA-binding proteins, subcellular localization of mRNAs^14^ and condition-dependent variations in the abundance and post-translational modifications of translation factors^15^ result in differences in protein output between mRNAs and between conditions. The codon usage of mRNAs has been shaped by the need to ensure appropriate co-translational protein folding^16^, and the interplay between codon bias and tRNA abundance mediates a translational code of cell proliferation^17^.

Ribosome profiling (also known as ribosome footprint sequencing or ribo-seq) is a technique that allows the protein output from virtually all expressed mRNAs to be accurately estimated^18,19^, enabling insights into the principles of translational control^20^. Its application in yeast has identified initiation as the major rate-limiting step of translation^19,21,22^, primarily influenced by features of the 5′UTR that affect ribosome recruitment and scanning^23,24^. Translation elongation rates also vary between mRNAs^19,25^, depending on the codon usage^26^, tRNA levels^11^, frequency of positively charged amino acids in the synthesized protein^19,27^, and availability of elongation factors^7,28^. The rate of elongation is globally reduced upon stress and short term energy depletion; this mechanism is thought to allow rapid resumption of translation once the conditions improve, without the need for ribosome dissociation and clearance of partial translation products^13,15,29^. Translation elongation rates can be estimated with both single molecule and transcriptome-wide approaches. Fluorescence imaging of reporters^30–35^ yielded values of ~3–5 aa/s^31^, similar to those obtained at meta-gene level from ribosome run-off experiments done in mouse embryonic stem cells (mESCs - 5.6 aa/s)^36^ and mouse organs (liver, kidney, and skeletal muscle - 6.8, 5.0, and 4.3 aa/s, respectively)^37^. On individual mRNAs, the translation elongation rates estimated by ribosome run-off in mESCs^36^ vary more than an order of magnitude, from «1 aa/s to 10–15 aa/s^38^. A similarly wide range was obtained in budding yeast cells with an orthogonal approach that combined measurements of ribosome density and protein synthesis rates^19^. The rates of translation initiation and elongation of individual mRNAs are highly correlated^19^. This could reflect a selection pressure for maintaining an adequate ribosome flow upon perturbations^29^, though the coupling mechanism(s) are still unclear.

**Fig. 1:**
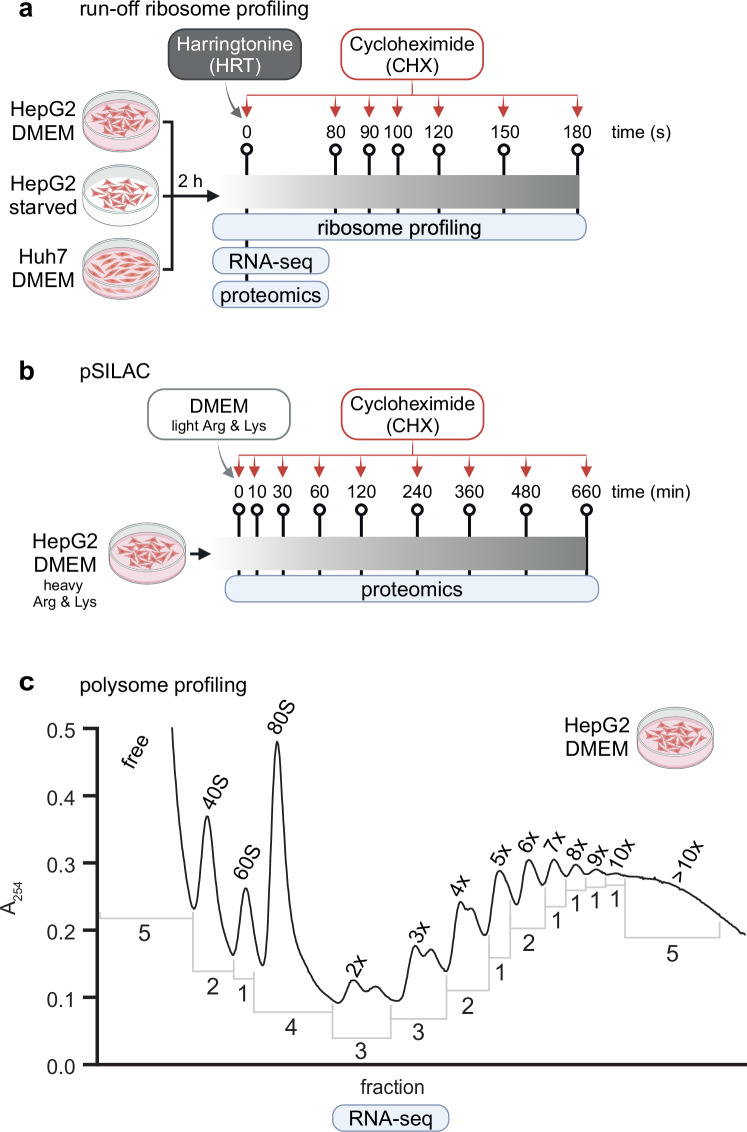
Experimental design. (**a**) Steady state and run-off ribosome profiling. Cells were grown to confluence in DMEM and then incubated for 2 h in DMEM (HepG2 and Huh7 cell lines) or in starvation medium (HBSS plus 4.5 g/l glucose, HepG2 cells). Then, harringtonine was added to arrest translation at initiation sites (time = 0) and, after the indicated time points, cycloheximide (CHX) was added to arrest overall translation. At time 0, RNA-seq and proteomics were also performed. (**b**) pSILAC. Cells were grown to confluence in DMEM containing stable isotopes of Arg and Lys and then shifted to DMEM containing light Arg and Lys. At the indicated time points, CHX was added and samples were collected. (**c**) Polysome profiling in HepG2 cells grown to confluence in DMEM. Absorbance peaks as a function of position in the ultracentrifuge tube are shown for a representative biological replicate. The number of ribosomes per mRNA is indicated above the polysome profile (2x = disome, 3x = trisome, etc.), while the numbers under the profile indicate the number of fractions that were combined to reconstruct individual peaks. Experiments were performed as three biological replicates except for the proteomics of Huh7 cells in DMEM at time 0 (performed in duplicate) and in starvation at time 0 (single value). Figure created with bioRender.com.

Being able to predict the protein output of individual mRNAs is important for a broad range of applications, from protein expression engineering to the design of mRNA vaccines. Machine learning models have started to emerge, trained on data from massively parallel reporter assays that measured randomized sequences^23,39^. However, compositional differences between random and evolved sequences limit the generality of the developed models. Therefore, more data on endogenous mRNAs is needed^40^. To fill this gap, we obtained steady-state and dynamic multi-omics data from human liver cancer cell lines, specifically (i) ribosome profiles of unperturbed cells and following translation initiation block (run-off ribosome profiling), (ii) protein synthesis rates estimated by pulsed stable isotope labeled amino acids in cell culture (pSILAC), and (iii) mean ribosome load on individual mRNAs determined by mRNA sequencing of polysome fractions (polysome profiling) (Fig. 1).

## Methods

### Cell culture

The HepG2 cell line originated from ATCC (#HB-8065) and was obtained from Dr. Salvatore Piscuoglio (DBM, Basel), while the Huh7 was purchased from Cell Lines Service (CLS, #300156). Unless otherwise indicated (see pSILAC), cells were cultured in Dulbecco’s Modified Eagle Medium - High glucose (4.5 g/l) (Sigma/Aldrich, Cat. No. D5796), containing 10% fetal calf serum, 4 mM L-glutamine, 1X Non-essential amino acid solution (ThermoFisher Scientific), 50 U/ml penicillin, 50 µg/ml streptomycin at 5% CO_2_ and 37 °C. Cells were passaged every 3-4 days. Absence of mycoplasma was verified using a PCR-based detection method as described in^41^. For standard conditions, cells were grown to achieve a 70–80% confluency and DMEM was replenished 3 h prior to cell collection. For starvation experiments (growth factor and amino acid starvation), after the 3 h of medium replenishment, cells were first washed once with starvation medium (Hank’s balanced salt solution plus calcium, magnesium (HBSS, Gibco, #14025-050), and adjusted to 4.5 g/l glucose) and then incubated for 2 h with the same starvation medium.

### Ribosome footprinting sequencing

For the ribosome run-off experiments, cells were treated with 4 µg/ml harringtonine (HRT) for 0, 80, 90, 100, 120, 150, and 180 seconds at 5% CO_2_ and 37 °C to arrest ribosomes at the translation start site before adding 100 μg/ml CHX to arrest elongating ribosomes. The cell collection and lysis, mRNA digestion, fractionation, and isolation, rRNA depletion and library preparation were performed as recently described^40^. Samples were sequenced on the NovaSeq 6000 system (Illumina) in the Genomics Facility Basel (Department of Biosystems Science and Engineering (D-BSSE), ETH Zürich). For data analysis, reads from fastq files were trimmed using Cutadapt to remove 5 nucleotides from the 5’ end (i.e. the G nucleotides that are added during library preparation). Ribosome footprints from Huh7 and starved HepG2 cells were sequenced in paired-end mode due to instrument availability, but we only used one of the mates for the analysis, as the paired reads yield redundant information. The FASTX-Toolkit (version 0.0.14, https://github.com/agordon/fastx_toolkit) was used to trim 3’ adapters, and reads were trimmed of low quality 3’ ends with fastq_quality_trimmer and the following parameters: minimum quality (-t) 20, offset for quality score (-Q) 33, minimum read length to keep (-l) 20. Reads were further filtered with fastq_quality_filter and parameters: minimum quality (-q) 20, minimum percentage of bases that must satisfy the quality criterion (-p) 90, and offset for quality score (-Q) 33. Following these pre-processing steps the fastq file was converted to fasta format with the fastq_to_fasta program from the same toolkit. Reads from the fasta file were aligned to the human ribosomal DNA repeat unit (Genbank accession U13369) using Segemehl version 0.2.0^42^ to filter out rRNA-derived reads. The reads that did not map to the rDNA with at least 90% accuracy (i.e. at most 10% nucleotide mismatches, insertions or deletions) were aligned to the longest annotated coding transcripts of protein-coding genes from the *Homo sapiens* genome assembly (GRCh38.105)^43^ using Segemehl version 0.2.0^42^ and the results were saved in a sam-formatted^44^ file. After the removal of multi-mappers the read length distribution was determined, and for each individual length, the offset of the P-site was estimated. The locations of P-sites inferred from all reads were tabulated to calculate the proportion of reads in each of the 3 possible reading frames. Count matrices were created from all reads mapping to individual transcripts and normalized for transcript length with a python script using the pysam package^45^, available from https://github.com/pysam-developers/pysam), yielding estimates of translation as transcripts per million (TPM) for further analysis.

### RNA-sequencing

Cell collection and lysis, RNA isolation, and library preparation using the stranded total RNA prep with Ribo-Zero plus (Illumina) was performed as in^40^. The RNA integrity number (RIN) of the samples was between 9.5 and 9.7. Samples were sequenced in a NovaSeq 6000 system (Illumina) in the Genomics Facility Basel (Department of Biosystems Science and Engineering, ETH Zürich). For the RNA-seq data analysis, paired-end reads from fastq files were processed using the ZARP workflow^46^, with default parameters. The reads were mapped to the transcriptome and the GRCh38.105 version of the *Homo sapiens* genome assembly. The parameter used for library type was ISR (inwards relative orientation of the reads, strand-specific protocol with read 1 coming from the reverse strand). The output of gene expression quantification by kallisto (version 0.46.2) was used for further analysis.

### Total proteomics sample preparation and analysis

#### Sample preparation

Protein quantification from lysates was performed by tryptophan fluorescence analysis (M-plex). Twenty-five µg of protein were diluted to a final volume of 25 µl in SDS-containing lysis buffer (5% SDS, 100 mM triethylammonium bicarbonate (TEAB) pH 8.5, 10 mM tris(2-carboxyethyl)phosphine (TCEP)). Then, 0.5 µl of 1 M iodoacetamide solution was added and the alkylation reaction was performed in the dark at 500 rpm for 30 min at 25 °C. Samples were acidified with 2.5 µl of 12% phosphoric acid (final concentration of 1.2%) and 165 µl S-Trap buffer (90% methanol, 100 mM TEAB pH 7.1) was added. Proteins were trapped in S-Trap micro columns (ProtiFi) and washed three times with 150 µl S-Trap buffer. After washing, protein digestion was performed by adding to the column 20 µl digestion buffer (20 mM TEAB pH 8.0) and 1 µg of sequencing‐grade modified trypsin (Promega). Samples were incubated for 1 h at 47 °C. After digestion, 40 µl of digestion buffer was added to the columns followed by centrifugation at 4’000 × g for 1 min. Then, 40 µl of 0.2% formic acid was added, followed again by centrifugation at 4’000 × g for 1 min. Lastly, to elute the peptides, 35 µl of acetonitrile + 0.2% formic acid solution was added to columns and they were centrifuged at 4’000 × g for 1 min. Eluted peptides were dried under vacuum. Dried peptides were resuspended in 20 µl of 0.1% aqueous formic acid. Peptide concentration was determined by UV-nanodrop and the peptide concentration set to 0.25 µg/µl by adding 0.1% aqueous formic acid. Then, 10x iRT peptide mix was added to each sample and mixed by vortexing.

#### LC-MS analysis

One μg of peptides were subjected to LC–MS/MS analysis using a Orbitrap Fusion Lumos Mass Spectrometer fitted with an EASY-nLC 1200 (both Thermo Fisher Scientific) and a custom-made column heater set to 60 °C. Peptides were resolved using a RP-HPLC column (75 μm × 36 cm) packed in-house with C18 resin (ReproSil-Pur C18–AQ, 1.9 μm resin; Dr. Maisch GmbH) at a flow rate of 0.2 μl/min. The following gradient was used for peptide separation: from 5% B to 12% B over 5 min to 35% B over 60 min to 50% B over 25 min to 95% B over 2 min followed by 18 min at 95% B. Buffer A was 0.1% formic acid in water and buffer B was 80% acetonitrile, 0.1% formic acid in water. The mass spectrometer was operated in DIA mode with a cycle time of 3 seconds. MS1 scans were acquired in the Orbitrap in centroid mode at a resolution of 120,000 FWHM (at 200 m/z), a scan range from 390 to 910 m/z, normalized AGC target set to 250% and maximum ion injection time mode set to 50 ms. MS2 scans were acquired in the Orbitrap in centroid mode at a resolution of 15,000 FWHM (at 200 m/z), precursor mass range of 400 to 900, quadrupole isolation window of 12 m/z with 1 m/z window overlap, a defined first mass of 120 m/z, normalized AGC target set to 2000% and a maximum injection time of 22 ms. Peptides were fragmented by HCD (Higher-energy collisional dissociation) with collision energy set to 33% and one microscan was acquired for each spectrum.

#### Protein identification and quantification

The generated raw files were searched against a database of human proteins (downloaded from Uniprot on February 2, 2022) and 392 commonly observed contaminants (total of 41504 protein sequences) using SpectroNaut (v18.6, Biognosys, Schlieren, CH) and default settings. Quantitative results obtained from the report file were further analyzed using the MSstats R package v.4.7.3 (refs. ^47,48^).

### pSILAC proteomics sample preparation and analysis

HepG2 cells were cultivated in SILAC-DMEM medium with high glucose (4.5 g/l) without L-leucine, L-arginine, L-lysine, L-methionine, phenol red, and sodium pyruvate (Molecular Dimensions, #MD 12-420) and supplemented with 10% FCS, 4 mM L-glutamine, 50 U/ml penicillin, 50 µg/ml streptomycin, 1x non essential amino acids (Gibco, #11140-050), L-leucine at a final concentration of 0.105 mg/ml, L-methionine at a final concentration of 0.03 mg/ml, L-lysine:2HCl (^13^C_6_, 99% ^15^N_2_, 99%, Lys8 (K), Eurisotop, #CNLM-291-H-0.1) at a final concentration of 0.146 mg/ml and L-arginine:HCL (^13^C_6_, 99% ^15^N_4_, 99%, Arg10 (R), Eurisotop, #CNLM-539-H-0.1) at a final concentration of 0.084 mg/ml. Cells were passed every 4 days for a total of 3 passes. Four days prior to the experiment, cells were seeded in 6 well plates at a density of 175’000 cells per well. The day of the experiment, confluent (80%) cells were briefly and gently rinsed twice, without detaching them, with 5 ml pre-warmed (37 °C) SILAC-DMEM-high glucose medium containing as above but with “light” L-lysine (final concentration of 0.146 mg/ml) and L-arginine (final concentration of 0.084 mg/ml) instead of the “heavy” versions. Then, 2.5 ml of this medium was added to cells and growth was resumed for the indicated times (0, 10, 30, 60, 120, 240, 360, and 480, and 660 min). CHX was added to the cells at a concentration of 100 µg/ml, medium was discarded and cells were washed with 500 µl PBS containing 100 µg/ml CHX. Another 500 µl PBS containing 100 µg/ml CHX were added and cells were scraped and collected into 1.5 ml tubes. Lysates were clarified by centrifugation at 1000 × g for 1 min at 4 °C, supernatant was removed, and cell pellets were snap frozen in liquid nitrogen and stored at −80 °C. Cells were lysed in 100 µl lysis buffer (5% SDS, 100 mM TEAB pH 8.5, 10 mM TCEP and 100 µg/ml CHX) using strong sonication for 10 min at 15 °C in a PIXUL sample sonicator (Active Motif). Protein samples were reduced for 10 minutes at 95 °C. Protein concentration from lysates was determined by tryptophan fluorescence analysis. A 0.4 M iodoacetamide solution was added to reach a final concentration of 15 mM and the cysteine residues were alkylated for 30 min at 25 °C in the dark. Sample aliquots containing 10 μg of total proteins were purified and digested with the SP3 approach^49^ using a Freedom Evo 100 liquid handling platform (Tecan Group Ltd., Männedorf, Switzerland). In brief, Speed Beads^TM^ (#45152105050250 and #65152105050250, GE Healthcare) were mixed 1:1, rinsed with water and diluted to a 8 μg/µl stock solution. Samples were adjusted to a final volume of 90 µl and 10 µl of beads stock solution was added to them. Proteins were bound to the beads by addition of 100 µl of 100% acetonitrile to the samples, which were then incubated for 8 min at RT with a gentle agitation (200 rpm). Samples were next placed on a magnetic rack and incubated for 5 minutes. Supernatants were removed and discarded. Beads were washed twice with 160 µl of 70% (v/v) ethanol and once with 160 of 100% acetonitrile. Samples were placed off the magnetic rack and 50 µl of digestion mix (10 ng/µl of trypsin in 50 mM TEAB) was added. Digestion was allowed to proceed for 12 h at 37 °C. Then, samples were placed back on the magnetic rack and incubated for 5 min. Supernatants containing peptides were collected and dried under vacuum. LC-MS analysis and protein identification and quantification steps were performed as above (see “Total proteomics sample preparation and analysis” section) with the only difference that Arg10 (R) and Lys8 (K) were additionally set as variable modifications.

#### Determination of protein synthesis rates

We selected the peptides corresponding to a single protein and containing either the heavy lysine or the heavy arginine amino acids. Next, we inferred the accumulation rate $k$ for a peptide by an exponential fit of signal intensities $I(t)={I}_{0}\ast {e}^{-{kt}}$, where ${I}_{0}$ is the peptide-specific intensity. Since the value of ${I}_{0}$ is not relevant for computing the rate and the function $I(t)$ is linear in ${I}_{0}$, we can simply project it out^50^ and therefore make the fit more stable. Peptide accumulation rates smaller than ${10}^{-5}$ per minute were considered to be spurious and therefore discarded. While intensity values for different peptides are not comparable, the accumulation rates are. Therefore, we averaged the accumulation rates of different peptides corresponding to the same protein and saved the average as well as the spread (maximum minus minimum value) as a crude measure of fluctuation. To obtain protein synthesis rates from the peptide accumulation rates we need to normalize by the overall abundance of proteins in this particular cell type. A prior study measured these abundances in triplicate^51^ and obtained very reproducible data. Thus, we pooled these replicates and divided the protein accumulation rates computed from each of our replicate experiments by the (pooled) estimated protein copy number per cell to arrive at the final quantity, the protein synthesis rate per cell.

### Polysome profiling

HepG2 cells were grown to 70–80% confluency and DMEM was replenished 3 h prior to cell collection. After medium replenishment, CHX was added to a final concentration of 200 μg/ml CHX to arrest elongating ribosomes. Cell collection and lysis were performed as recently described^40^. A total of 3 A_260_ units was loaded onto a 20 to 60% sucrose gradient. Ultracentrifugation was carried out with a SW41Ti rotor at 210’100 × g for 3 h at 4 °C. Forty different fractions (270 μl each) were collected and all fractions corresponding to the same ribosomal peak were pooled into a single tube (Fig. 1). SIRV-Set 3 spike-in (Lexogen) RNA was added to the pooled fractions according to product instructions. RNA was then purified by acid phenol: chloroform: isoamyl alcohol (25: 24: 1). RNA precipitation was carried out by adding 2.5 volumes of ethanol and 2 μl of GlycoBlue (Thermo Fisher Scientific) as coprecipitant followed by chilling overnight at −80 °C. The samples were then spun down at 20’000 × g for 30 min, washed with 75% ethanol and then the RNA was reconstituted in 20 μl of nuclease-free water (Thermo Fisher Scientific). Finally, the RNA was cleaned up with a Zymo RNA clean and concentrator (RCC) kit, incorporating an on-column DNA digestion step to remove DNA contamination. Ribosomal RNA was depleted with the Truseq Stranded Total RNA LP Gold kit (Illumina) by using 100 ng RNA as input and Truseq RNA UD Indexes v2 for i5/i7. Libraries were sequenced in a NovaSeq 6000 system (Illumina) on 1 lane of an S4 flow cell (XP mode)  in the Genomics Facility Basel resulting in paired-end reads of 101 bp length.

### Computation of average ribosome load per transcript

The polysome fraction RNA-seq samples were analyzed individually, each fraction and replicate, with ZARP^46^, augmenting the human genome annotation from ENSEMBL (GRCh38.105) with the spike-in sequences SIRV-Set 3 Normal (https://www.lexogen.com/sirvs/download/). We extracted reads mapped to exons with Rsubread^52^ and calculated TPM values as proxies for the representation of each expressed gene in each fraction. The computation of the mean ribosome load according to ref. ^23^ is naturally limited by the highest resolved polysome (10 in our case), which could lead to substantial imprecision for long transcripts and high expression levels. However, under the recently verified assumption of low density of ribosomes per transcript^53^ we expect that the number of ribosomes per transcript follows a Poisson distribution with parameter $\lambda ,{p}({k;}\lambda )=\frac{{\lambda }^{k}}{k!}{e}^{-\lambda }$, where $k$ is the number of ribosomes translating a given mRNA. $\lambda =\, < k >$ is the mean ribosome load per mRNA that we estimate as follows. We denote the total number of spike-in transcripts by $S$, the same across polysome fractions $k=0\,({free}),\,0\,(40S),\,1\,(80S),\,2\,({disome})$, etc., because an equal amount of spike-in mixture was added to each fraction. From the definition of the total TPM of the spike-ins in polysome fraction $k$, ${{TPM}}_{S}^{k}=\frac{S}{S+{M}^{k}}\ast {10}^{6}$, where ${M}^{k}$ is the total number of endogenous mRNA molecules in fraction $k$, we have that $S+{M}^{k}=\frac{S\ast {10}^{6}}{{{TPM}}_{s}^{k}}$. If we focus in a single mRNA type, $i$, its TPM in fraction $k$ is given by ${{TPM}}_{i}^{k}=\frac{{M}_{i}^{k}}{S+{M}^{k}}\ast {10}^{6}=\frac{{M}_{i}^{k}\,\ast \,{{TPM}}_{i}^{k}}{S}$, where ${M}_{i}^{k}$ is the count of mRNAs of type $i$ in ribosome fraction $k$. Thus, the absolute number of molecules of mRNA $i$ in fraction $k$ is ${M}_{i}^{k}=\frac{{{TPM}}_{i}^{k}\ast S}{{{TPM}}_{s}^{k}}$ and by normalizing to the total ${M}_{i}^{k}$ we obtain the probability ${p}_{i}^{k}$ to find a molecule of type $i$ in fraction $k$. This is equivalent to the probability distribution of the number of ribosomes $k$ translating transcripts of type $i$, and by fitting a Poisson distribution to the calculated ${p}_{i}^{k}$’s we obtain the mean ribosome load on each transcript type. As we do not have reliable error estimates of $p({k;}\lambda )$ we cannot compute meaningful ${\chi }^{2}$ values. However, the returned ${\chi }^{2}$ give some information about the quality of the Poissonian fit for one gene relative to others.

### Computation of codon enrichments

The codon enrichments are estimated as described by Legrand & Tuorto^54^, namely as the ratio between the observed and expected codon coverage, where the expected coverage is the codon usage across the translatome. The expected usage of codon *c* is calculated as the average codon usage in the coding regions, weighted by the number of footprints per coding region. The observed usage of codon *c* is estimated as the proportion of footprints that have *c* at their A-site (located at a footprint length-dependent distance from the 5’ end of the footprint) across all coding regions. To avoid biases that could be introduced by sample preparation^55^, the first 15 codons from the start site and the last 5 codons from the stop site are excluded from the analysis.

## Data Records

**Fig. 2:**
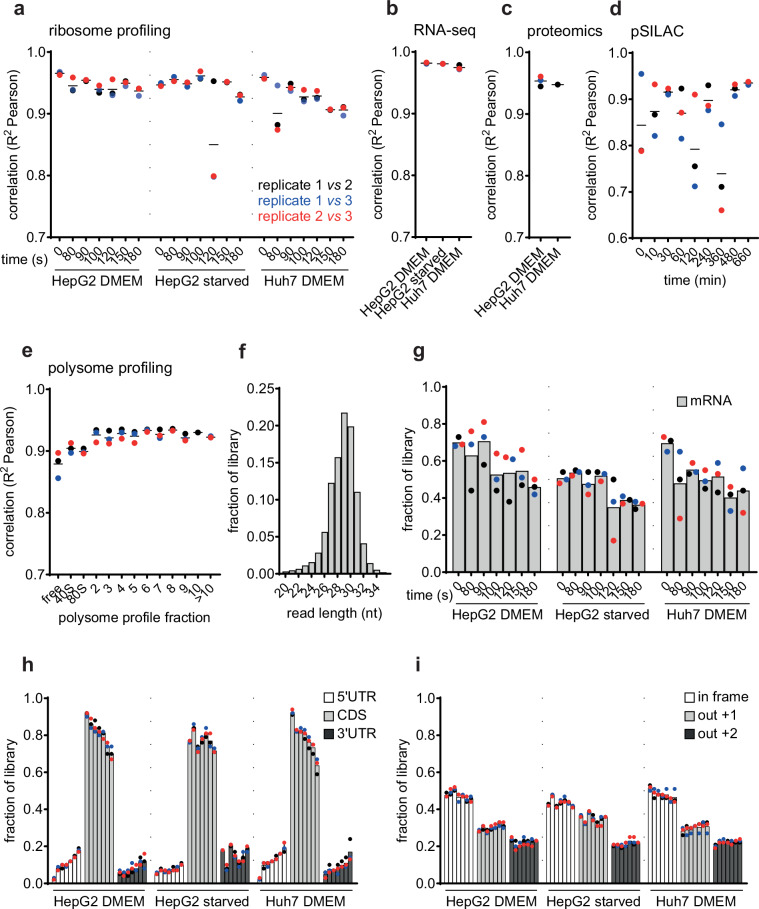
Assessment of experimental reproducibility. (**a**) Pairwise Pearson correlation coefficients of three biological replicates of mRNA-length normalized ribosome footprints (TPM) from run-off ribosome profiling experiments. (**b**) Pairwise Pearson correlation coefficients of three biological replicates of mRNA expression levels (TPM from RNA-seq), obtained from time 0 (immediately after harringtonine treatment). (**c**) Pairwise Pearson correlation coefficients of biological replicates of peptide intensities measured at time 0 of the proteomics experiments (see Fig. 1a). (**d**) Pairwise Pearson correlation coefficients of heavy peptide intensities from the pSILAC time course experiment. (**e**) Pairwise Pearson correlation coefficients of mRNA abundance (TPM) in each sucrose fraction of 3 biological replicates of polysome profiling experiments. (**f**) Length distribution of ribosome footprints from all samples. (**g**) Fraction of reads mapping to mRNAs in run-off ribosome profiling experiments. (**h**) Relative proportion of reads mapping to 5′UTR, CDS, or 3′UTR regions of the mRNAs in run-off ribosome profiling experiments. (**i**) Proportion of reads mapping to the three possible frames in ribosome run-off experiments. The P-site offsets were calculated for each individual read length, the reads were tabulated according to the respective offsets, and the relative proportion of reads associated with each possible reading frame was calculated. All panels have been created with the software GraphPad Prism 10.

**Fig. 3:**
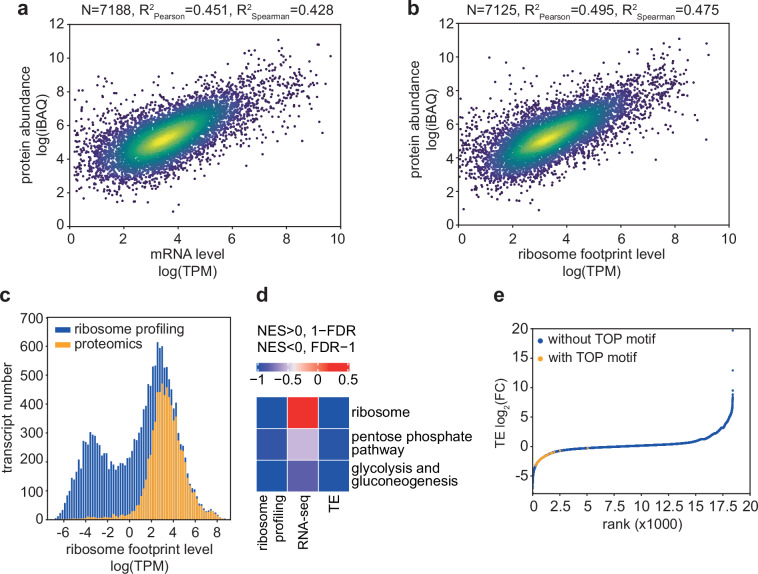
Consistency of gene expression estimates. (**a**) Correlation of protein abundances, measured by iBAQ (log intensity values), and mRNA levels (log TPM) measured by RNA-seq. (**b**) Correlation of protein abundances, measured by iBAQ (log intensity values), and ribosome footprints levels (log TPM) measured by ribosome profiling. (**c**) Histogram showing the distribution of abundances in the translatome of transcripts detected by ribosome profiling (blue) compared to transcripts whose corresponding proteins were measured by proteomics (orange). (**d**) Gene Set Enrichment Analysis showing functional categories (KEGG) of genes with significant change at mRNA level (RNA-seq), translatome level (ribosome profiling) or translation efficiency (TE). (**e**) Cumulative density of log2 fold-changes in TE per mRNA upon starvation. Highlighted in orange are the TOP motif-containing mRNAs.

**Fig. 4:**
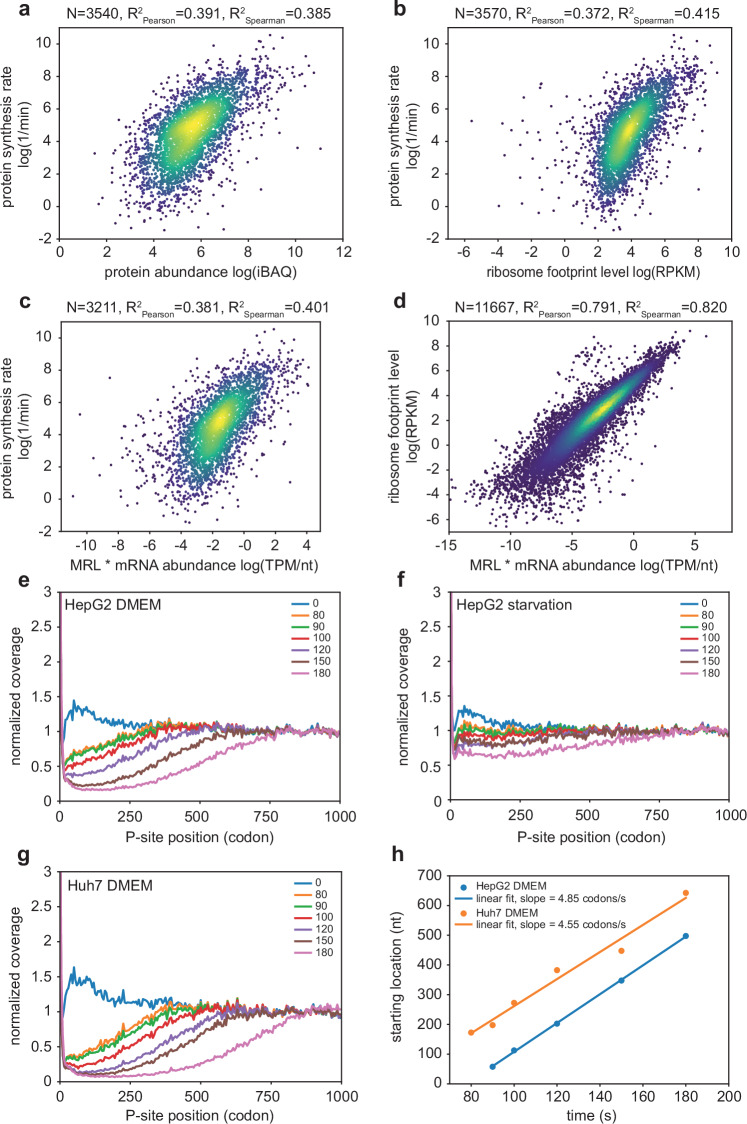
Translation parameters estimated with different approaches. (**a**) Correlation between steady-state protein abundance and protein synthesis rate measured by pSILAC. (**b**) Relationship between ribosome footprints per mRNA (measured in reads per kilobase per million, RPKM) and protein synthesis rate. (**c**) Correlation between the mean ribosome load per nucleotide and the protein synthesis rate per transcript. (**d**) Correlation of ribosome density estimated from ribo-seq (RPKM) and from the measurements of mean ribosome load per nucleotide. (**e-g**) Metagene analyses of run-off elongation in HepG2 cells growing in DMEM (**e**), under starvation (**f**) or Huh7 cells growing in DMEM (**g**). Replicates of ribosome profiling samples were pooled, the coverage was averaged within windows of 5 codons and the ratio relative to the coverage in the region of 800–1000 codons is shown (normalized coverage). Each curve corresponds to a defined time point after the treatment with harringtonine (indicated in the legend). (**h**) Starting locations plotted against the respective runoff times for the HepG2 and Huh7 datasets. According to^36^, the starting location is where the normalized coverage reaches 0.5 for the first time when traversing the curve from start to end of metagene. A global estimate for the elongation rate can be read off as the slope of a linear fit.

Sequencing data was uploaded to Sequence Read Archive database (SRA) under accession number SRP530258 and can be accessed with the permanent link http://identifiers.org/insdc.sra:SRP530258^56^. All mass spectrometry files (see Supplementary Table S1 for legend) associated with this manuscript are accessible at MassIVE (https://massive.ucsd.edu) under accession number MSV000096514, with the following permanent link http://identifiers.org/massive:MSV000096514^57^. The Zenodo record^58^ contains (i) plot scripts of Figs. 2, 3, and 4, (ii) BigWig files for all the ribosome footprinting libraries analyzed in this study, (iii) the FASTA file that was used as reference transcriptome, (iv) the master table (and its legend) for details about the -omics data, and (v) the codon enrichment values table.

## Technical Validation

### Reproducibility

We assessed the reproducibility of ribosome profiling datasets by calculating the Pearson correlation coefficients of mRNA length-normalized ribosome footprints (as transcripts-per-million, TPM) in pairs of samples. Pearson R^2^ values were very high, ~ 0.95 (Fig. 2a), with only 2 samples (replicate 3 of HepG2 starvation at 120 s and replicate 2 of Huh7 at 80 s) being slightly less well correlated with their biological replicates (R^2^ Pearson ~ 0.8 for replicate 3 vs. 1 or 2 of HepG2 120 s and R^2^ Pearson ~ 0.9 for replicate 2 vs 1 or 3 of Huh7 80 s). All the numbers are in line with the expected reproducibility of ribosome profiling experiments^40^. We were unable to identify a specific factor accounting for the variability of those specific samples; it seems likely that ensuring high reproducibility in the preparation of such a large number of samples remains challenging. The inter-replicate Pearson correlation coefficients of RNA-seq-based measurements of mRNA levels (Fig. 2b), mass spectrometry-based measurements of protein expression levels (Fig. 2c), heavy peptide intensities measured by pSILAC (Fig. 2d), and mRNA abundance in polysome fractions (Fig. 2e) were also high. The highest variability was observed among measurements of peptide intensities, with R^2^ values ~0.7 for some of the replicates (Fig. 2d). Altogether, these results demonstrate the high reproducibility of individual data types.

### Technical rigor assessment

To further illustrate the quality of the data, we computed a few other broadly-adopted measures of quality for specific data types. The percentage of uniquely mapped RNA-seq reads was between 72–82%, which is in the expected range^59^. The ribosome protected fragments (RPFs) had the expected length, 27 to 31 nucleotides, with the mode at 29–30 nucleotides^13^ (Fig. 2f), and the majority mapped to mRNAs (Fig. 2g), within coding regions (CDS, 65 to 95% of reads in a sample, Fig. 2h). The proportion of CDS-mapped decreased with increasing duration of the harringtonine treatment, as expected from ribosomes running off the mRNA (Fig. 2i). The ribosome footprints also showed the expected three nucleotide periodicity of P-sites (Fig. 2h). Altogether, these measurements indicate the reliability of the ribosome footprint data.

### Consistency of gene expression estimates obtained with different technologies

We next checked the consistency of gene expression estimates, determined at the protein level by mass spectrometry (intensity-based absolute quantification, iBAQ), at mRNA level by RNA-seq (Fig. 3a), and at the level of translation by ribosome footprinting (Fig. 3b). The mRNA-length normalized ribosome footprints explained slightly more of the variance in protein level than mRNAs, ~50% compared to ~45%, though as expected, mass spectrometry mainly captured highly-translated proteins (Fig. 3c). As translation is strongly remodeled by starvation^60,61^, we further assessed whether our experiments capture this effect, obtaining ribosome footprints from HepG2 cells cultured for 2 h in HBSS plus glucose medium (see Methods). Using RNA-seq and ribosome profiling data to estimate the change in the translation efficiency of mRNAs (TE = TPM from ribosome profiling divided by TPM from RNA-seq) we found that 89 out of 92 TOP motif-containing genes are strongly downregulated in TE upon starvation (Fig. 3d). TOP motif genes encode essential protein synthesis factors, which are the primary targets of translational control under stress^62^. Because most of these factors are ribosomal proteins^63^, the KEGG category “ribosome“ appears as strongly repressed in Gene Set Enrichment Analysis^64^. Also consistently, energy metabolism-related pathways are downregulated (Fig. 3d,e). Thus, our multi-omics measurements are highly consistent with each other, reflecting the expected response of translation to specific stimuli.

### Consistency of translation parameters estimated with different approaches

The abundance of proteins in a cell is determined by their rates of synthesis and degradation. The variation in degradation rates among proteins is more limited than the variation in synthesis rates^4^, which is why the protein levels measured by iBAQ and the protein synthesis rates estimated from pSILAC show substantial correlation, R^2^ Pearson = 0.391 (Fig. 4a). The CDS length-normalized number of ribosome footprints (measured as reads per kilobase of coding region per million reads, RPKM) shows a similar correlation with the protein synthesis rate, R^2^ Pearson = 0.372 (Fig. 4b). In addition, as mentioned before, ribosome profiling provides more extensive data - here we identified RPFs on 20’725 genes - than protein quantification by mass spectrometry - 3’589 proteins measured with pSILAC, all but 19 of which were also covered by ribosome profiling. Similar measurements in budding yeast yielded a similar correlation coefficient^19^. Also similar to yeast, the HepG2 mRNAs with high coverage by ribosomes but lower than expected protein synthesis rate (top right side of the plot) encode ribosomal proteins, which interact with the peptide exit tunnel of the ribosome via positive charges^19,65^.

Another measure used in large scale studies of translation is the mean ribosome load per mRNA^23^, calculated from the relative abundance of individual mRNA species in fractions of the polysome gradient corresponding to one, two, etc. ribosomes per mRNA. It is expected that the number of mRNAs and the mean ribosome load determine the protein synthesis rate and indeed, the product of MRL and mRNA abundance (estimated as TPM) equally predictive of the protein synthesis rate as level of ribosome footprints (R^2^ Pearson = 0.381, Fig. 4c compared to Fig. 3b). The ribosome and polysome-profile based measures of translation yield very similar numbers up to a scaling factor (R^2^ Pearson = 0.791, Fig. 4d).

Finally, the metagene analysis of ribosome run-off data (Figs. 4e to 4h) yielded an average translation elongation rate of 4.55 and 4.85 aa/s for HepG2 cells and Huh7 cells, respectively (Fig. 4h), very similar to values reported in prior studies^36–38^. The pattern of RPF coverage in the starvation experiment indicates severely impaired translation, with very small differences between time points of the run-off experiment, which is why we did not attempt to estimate the mean rate of elongation in this experiment (Fig. 4f). Codon enrichment values, reflecting their decoding speed^54^ are given in the zenodo record^58^.

The extent to which the ribosome flow has been optimized on individual eukaryotic mRNAs so as to enable accurate protein folding and achieve specific protein levels, as well as the underlying mechanisms are still unclear. Studies combining mathematical models with measurements of translation indicate that in yeast, the translation parameters have been optimized to ensure ribosome flow and minimize the overall cost of translation^19,21,66^. While the design principles are expected to be conserved, direct evidence from human cells is more scarce. We expect that our multi-faceted data on ribosome flow, ribosome load and protein production will shed light on the main parameters of translation of individual human mRNAs. In particular, it will be interesting to compare estimates of translation elongation obtained from ribosome runoff data with the predictions of the model proposed by Erdmann-Pham *et al*.^21^ based on ribosome profiling data.

## Supplementary information

Supplementary Table S1

## Data Availability

Sequencing data was uploaded to SRA under accession number SRP530258 (http://identifiers.org/insdc.sra:SRP530258)^56^. Mass spectrometry files (see Supplementary Table S1 for legend) are accessible at MassIVE under accession number MSV000096514 (http://identifiers.org/massive:MSV000096514)^57^. The bigWig files associated with the ribosome footprinting experiments, the master data table, and the codon enrichment values table are provided as part of the zenodo record (10.5281/zenodo.15858175)^58^.

## Code availability

The analysis scripts for protein synthesis rates from pSILAC and average ribosome loads from polysome profiling are deployed in a github repository [https://github.com/zavolanlab/mapping-translation-rates]. Plot scripts in python and R can be found in the zenodo archive^58^.

## References

[1] Sonneveld, S., Verhagen, B. M. P. & Tanenbaum, M. E. Heterogeneity in mRNA Translation. *Trends Cell Biol.* **30**, 606–618 (2020). 32461030 10.1016/j.tcb.2020.04.008

[2] Buccitelli, C. & Selbach, M. mRNAs, proteins and the emerging principles of gene expression control. *Nat. Rev. Genet.* **21**, 630–644 (2020). 32709985 10.1038/s41576-020-0258-4

[3] Liu, Y., Beyer, A. & Aebersold, R. On the Dependency of Cellular Protein Levels on mRNA Abundance. *Cell* **165**, 535–550 (2016). 27104977 10.1016/j.cell.2016.03.014

[4] Schwanhäusser, B. *et al*. Global quantification of mammalian gene expression control. *Nature* **473**, 337–342 (2011). 21593866 10.1038/nature10098

[5] Bourke, A. M., Schwarz, A. & Schuman, E. M. De-centralizing the Central Dogma: mRNA translation in space and time. *Mol. Cell* **83**, 452–468 (2023). 36669490 10.1016/j.molcel.2022.12.030

[6] Tahmasebi, S., Khoutorsky, A., Mathews, M. B. & Sonenberg, N. Translation deregulation in human disease. *Nat. Rev. Mol. Cell Biol.* **19**, 791–807 (2018). 30038383 10.1038/s41580-018-0034-x

[7] Robichaud, N., Sonenberg, N., Ruggero, D. & Schneider, R. J. Translational Control in Cancer. Cold Spring Harb. Perspect. Biol. 11 (2019). 10.1101/cshperspect.a032896 PMC6601465 29959193

[8] Saba, J. A., Liakath-Ali, K., Green, R. & Watt, F. M. Translational control of stem cell function. *Nat. Rev. Mol. Cell Biol.* **22**, 671–690 (2021). 34272502 10.1038/s41580-021-00386-2

[9] Teixeira, F. K. & Lehmann, R. Translational Control during Developmental Transitions. Cold Spring Harb. Perspect. Biol. 11 (2019). 10.1101/cshperspect.a032987 PMC6546043 30082467

[10] Harnett, D. *et al*. A critical period of translational control during brain development at codon resolution. *Nat. Struct. Mol. Biol.* **29**, 1277–1290 (2022). 36482253 10.1038/s41594-022-00882-9 PMC9758057

[11] Gingold, H. *et al*. A dual program for translation regulation in cellular proliferation and differentiation. *Cell* **158**, 1281–1292 (2014). 25215487 10.1016/j.cell.2014.08.011

[12] Wolf, T. *et al*. Dynamics in protein translation sustaining T cell preparedness. *Nat. Immunol.* **21**, 927–937 (2020). 32632289 10.1038/s41590-020-0714-5 PMC7610365

[13] Wu, C. C.-C., Zinshteyn, B., Wehner, K. A. & Green, R. High-Resolution Ribosome Profiling Defines Discrete Ribosome Elongation States and Translational Regulation during Cellular Stress. *Mol. Cell* **73**, 959–970.e5 (2019). 30686592 10.1016/j.molcel.2018.12.009 PMC6411040

[14] Jan, C. H., Williams, C. C. & Weissman, J. S. Principles of ER cotranslational translocation revealed by proximity-specific ribosome profiling. *Science* **346**, 1257521 (2014). 25378630 10.1126/science.1257521 PMC4285348

[15] Proud, C. G. Phosphorylation and Signal Transduction Pathways in Translational Control. Cold Spring Harb. Perspect. Biol. 11 (2019). 10.1101/cshperspect.a033050 PMC6601458 29959191

[16] Yu, C.-H. *et al*. Codon usage influences the local rate of translation elongation to regulate co-translational protein folding. *Mol. Cell* **59**, 744–754 (2015). 26321254 10.1016/j.molcel.2015.07.018 PMC4561030

[17] Gingold, H. & Pilpel, Y. Determinants of translation efficiency and accuracy. *Mol. Syst. Biol.* **7**, 481 (2011). 21487400 10.1038/msb.2011.14 PMC3101949

[18] Ingolia, N. T., Ghaemmaghami, S., Newman, J. R. S. & Weissman, J. S. Genome-wide analysis *in vivo* of translation with nucleotide resolution using ribosome profiling. *Science* **324**, 218–223 (2009). 19213877 10.1126/science.1168978 PMC2746483

[19] Riba, A. *et al*. Protein synthesis rates and ribosome occupancies reveal determinants of translation elongation rates. *Proc. Natl. Acad. Sci. USA.* **116**, 15023–15032 (2019). 31292258 10.1073/pnas.1817299116 PMC6660795

[20] Hershey, J. W. B., Sonenberg, N. & Mathews, M. B. Principles of Translational Control. Cold Spring Harb. Perspect. Biol. 11 (2019). 10.1101/cshperspect.a032607 PMC6719596 29959195

[21] Erdmann-Pham, D. D., Dao Duc, K. & Song, Y. S. The Key Parameters that Govern Translation Efficiency. *Cell Syst* **10**, 183–192.e6 (2020). 31954660 10.1016/j.cels.2019.12.003 PMC7047610

[22] Shah, P., Ding, Y., Niemczyk, M., Kudla, G. & Plotkin, J. B. Rate-limiting steps in yeast protein translation. *Cell* **153**, 1589–1601 (2013). 23791185 10.1016/j.cell.2013.05.049 PMC3694300

[23] Sample, P. J. *et al*. Human 5′ UTR design and variant effect prediction from a massively parallel translation assay. *Nat. Biotechnol.* **37**, 803–809 (2019). 31267113 10.1038/s41587-019-0164-5 PMC7100133

[24] Niederer, R. O., Rojas-Duran, M. F., Zinshteyn, B. & Gilbert, W. V. Direct analysis of ribosome targeting illuminates thousand-fold regulation of translation initiation. *Cell Syst.* **13**, 256–264.e3 (2022). 35041803 10.1016/j.cels.2021.12.002 PMC8930539

[25] Weinberg, D. E. *et al*. Improved Ribosome-Footprint and mRNA Measurements Provide Insights into Dynamics and Regulation of Yeast Translation. *Cell Rep.* **14**, 1787–1799 (2016). 26876183 10.1016/j.celrep.2016.01.043 PMC4767672

[26] Liu, Y. A code within the genetic code: codon usage regulates co-translational protein folding. *Cell Commun. Signal.* **18**, 145 (2020). 32907610 10.1186/s12964-020-00642-6 PMC7488015

[27] Lu, J. & Deutsch, C. Electrostatics in the ribosomal tunnel modulate chain elongation rates. *J. Mol. Biol.* **384**, 73–86 (2008). 18822297 10.1016/j.jmb.2008.08.089 PMC2655213

[28] Neelagandan, N., Lamberti, I., Carvalho, H. J. F., Gobet, C. & Naef, F. What determines eukaryotic translation elongation: recent molecular and quantitative analyses of protein synthesis. *Open Biol.* **10**, 200292 (2020). 33292102 10.1098/rsob.200292 PMC7776565

[29] Browne, G. J. & Proud, C. G. Regulation of peptide-chain elongation in mammalian cells: Control of translation elongation. *Eur. J. Biochem.* **269**, 5360–5368 (2002). 12423334 10.1046/j.1432-1033.2002.03290.x

[30] Tanenbaum, M. E., Gilbert, L. A., Qi, L. S., Weissman, J. S. & Vale, R. D. A protein-tagging system for signal amplification in gene expression and fluorescence imaging. *Cell* **159**, 635–646 (2014). 25307933 10.1016/j.cell.2014.09.039 PMC4252608

[31] Yan, X., Hoek, T. A., Vale, R. D. & Tanenbaum, M. E. Dynamics of Translation of Single mRNA Molecules In. *Vivo. Cell* **165**, 976–989 (2016). 27153498 10.1016/j.cell.2016.04.034 PMC4889334

[32] Wang, C., Han, B., Zhou, R. & Zhuang, X. Real-Time Imaging of Translation on Single mRNA Transcripts in Live Cells. *Cell* **165**, 990–1001 (2016). 27153499 10.1016/j.cell.2016.04.040 PMC4905760

[33] Mateju, D. *et al*. Single-Molecule Imaging Reveals Translation of mRNAs Localized to Stress Granules. *Cell* **183**, 1801–1812.e13 (2020). 33308477 10.1016/j.cell.2020.11.010

[34] Wilbertz, J. H. *et al*. Single-Molecule Imaging of mRNA Localization and Regulation during the Integrated Stress Response. *Mol. Cell* **73**, 946–958.e7 (2019). 30661979 10.1016/j.molcel.2018.12.006

[35] Morisaki, T. *et al*. Real-time quantification of single RNA translation dynamics in living cells. *Science* **352**, 1425–1429 (2016). 27313040 10.1126/science.aaf0899

[36] Ingolia, N. T., Lareau, L. F. & Weissman, J. S. Ribosome profiling of mouse embryonic stem cells reveals the complexity and dynamics of mammalian proteomes. *Cell* **147**, 789–802 (2011). 22056041 10.1016/j.cell.2011.10.002 PMC3225288

[37] Gerashchenko, M. V., Peterfi, Z., Yim, S. H. & Gladyshev, V. N. Translation elongation rate varies among organs and decreases with age. *Nucleic Acids Res.* **49**, e9 (2021). 33264395 10.1093/nar/gkaa1103 PMC7826258

[38] Dana, A. & Tuller, T. Determinants of translation elongation speed and ribosomal profiling biases in mouse embryonic stem cells. *PLoS Comput. Biol.* **8**, e1002755 (2012). 23133360 10.1371/journal.pcbi.1002755 PMC3486846

[39] Cuperus, J. T. *et al*. Deep learning of the regulatory grammar of yeast 5′ untranslated regions from 500,000 random sequences. *Genome Res.* **27**, 2015–2024 (2017). 29097404 10.1101/gr.224964.117 PMC5741052

[40] Schlusser, N., González, A., Pandey, M. & Zavolan, M. Current limitations in predicting mRNA translation with deep learning models. *Genome Biol.* **25**, 227 (2024). 39164757 10.1186/s13059-024-03369-6 PMC11337900

[41] Uphoff, C. C. & Drexler, H. G. Comparative PCR analysis for detection of mycoplasma infections in continuous cell lines. *In Vitro Cell. Dev. Biol. Anim.* **38**, 79–85 (2002). 11928999 10.1290/1071-2690(2002)038<0079:CPAFDO>2.0.CO;2

[42] Otto, C., Stadler, P. F. & Hoffmann, S. Lacking alignments? The next-generation sequencing mapper segemehl revisited. *Bioinformatics* **30**, 1837–1843 (2014). 24626854 10.1093/bioinformatics/btu146

[43] Martin, F. J. *et al*. Ensembl 2023. *Nucleic Acids Res.* **51**, D933–D941 (2023). 36318249 10.1093/nar/gkac958 PMC9825606

[44] Li, H. *et al*. The Sequence Alignment/Map format and SAMtools. *Bioinformatics* **25**, 2078–2079 (2009). 19505943 10.1093/bioinformatics/btp352 PMC2723002

[45] Danecek, P. **et al**. Twelve years of SAMtools and BCFtools. Gigascience 10 (2021). 10.1093/gigascience/giab008 PMC7931819 33590861

[46] Katsantoni, M. *et al*. ZARP: A user-friendly and versatile RNA-seq analysis workflow. *F1000Res.* **13**, 533 (2024).

[47] Kohler, D. *et al*. MSstats Version 4.0: Statistical Analyses of Quantitative Mass Spectrometry-Based Proteomic Experiments with Chromatography-Based Quantification at Scale. *J. Proteome Res.* **22**, 1466–1482 (2023). 37018319 10.1021/acs.jproteome.2c00834 PMC10629259

[48] Choi, M. *et al*. MSstats: an R package for statistical analysis of quantitative mass spectrometry-based proteomic experiments. *Bioinformatics* **30**, 2524–2526 (2014). 24794931 10.1093/bioinformatics/btu305

[49] Hughes, C. S. *et al*. Single-pot, solid-phase-enhanced sample preparation for proteomics experiments. *Nat. Protoc.* **14**, 68–85 (2019). 30464214 10.1038/s41596-018-0082-x

[50] O’Leary, D. P. & Rust, B. W. Variable projection for nonlinear least squares problems. *Comput. Optim. Appl.* **54**, 579–593 (2012).

[51] Wiśniewski, J. R., Vildhede, A., Norén, A. & Artursson, P. In-depth quantitative analysis and comparison of the human hepatocyte and hepatoma cell line HepG2 proteomes. *J Proteomics* **136**, 234–247 (2016). 26825538 10.1016/j.jprot.2016.01.016

[52] Liao, Y., Smyth, G. K. & Shi, W. The R package Rsubread is easier, faster, cheaper and better for alignment and quantification of RNA sequencing reads. *Nucleic Acids Res.* **47**, e47 (2019). 30783653 10.1093/nar/gkz114 PMC6486549

[53] Tomuro, K. *et al*. Calibrated ribosome profiling assesses the dynamics of ribosomal flux on transcripts. *Nat. Commun.* **15**, 7061 (2024). 39187487 10.1038/s41467-024-51258-0 PMC11347596

[54] Legrand, C. & Tuorto, F. RiboVIEW: a computational framework for visualization, quality control and statistical analysis of ribosome profiling data. *Nucleic Acids Res.* **48**, e7 (2020). 31777932 10.1093/nar/gkz1074 PMC6954398

[55] Hussmann, J. A., Patchett, S., Johnson, A., Sawyer, S. & Press, W. H. Understanding biases in ribosome profiling experiments reveals signatures of translation dynamics in yeast. *PLoS Genet.* **11**, e1005732 (2015). 26656907 10.1371/journal.pgen.1005732 PMC4684354

[56] EMBL-EBI. ENA Browser. http://identifiers.org/insdc.sra:SRP530258.

[57] Schmidt, A. MassIVE. http://identifiers.org/massive:MSV000096514.

[58] Schlusser, N. & Ataman, M. Plot scripts for “Multi-omic assessment of mRNA translation dynamics in liver cancer cell lines”. *Zenodo*10.5281/zenodo.15858175 (2025).10.1038/s41597-025-05861-540885748

[59] Conesa, A. *et al*. A survey of best practices for RNA-seq data analysis. *Genome Biol.* **17**, 13 (2016). 26813401 10.1186/s13059-016-0881-8 PMC4728800

[60] Leprivier, G. *et al*. The eEF2 Kinase Confers Resistance to Nutrient Deprivation by Blocking Translation Elongation. *Cell* **153**, 1064–1079 (2013). 23706743 10.1016/j.cell.2013.04.055 PMC4395874

[61] Darnell, A. M., Subramaniam, A. R. & O’Shea, E. K. Translational Control through Differential Ribosome Pausing during Amino Acid Limitation in Mammalian Cells. *Mol Cell* **71**, 229–243.e11 (2018). 30029003 10.1016/j.molcel.2018.06.041 PMC6516488

[62] Thoreen, C. C. *et al*. A unifying model for mTORC1-mediated regulation of mRNA translation. *Nature* **485**, 109–113 (2012). 22552098 10.1038/nature11083 PMC3347774

[63] Cockman, E., Anderson, P. & Ivanov, P. TOP mRNPs: Molecular Mechanisms and Principles of Regulation. Biomolecules 10 (2020). 10.3390/biom10070969 PMC7407576 32605040

[64] Subramanian, A. *et al*. Gene set enrichment analysis: a knowledge-based approach for interpreting genome-wide expression profiles. *Proc. Natl. Acad. Sci. USA.* **102**, 15545–15550 (2005). 16199517 10.1073/pnas.0506580102 PMC1239896

[65] Dao Duc, K. & Song, Y. S. The impact of ribosomal interference, codon usage, and exit tunnel interactions on translation elongation rate variation. *PLoS Genet.* **14**, e1007166 (2018). 29337993 10.1371/journal.pgen.1007166 PMC5786338

[66] Szavits-Nossan, J. & Ciandrini, L. Inferring efficiency of translation initiation and elongation from ribosome profiling. *Nucleic Acids Res.* **48**, 9478–9490 (2020). 32821926 10.1093/nar/gkaa678 PMC7515720

